# Effects of Coaxial Nozzle’s Inner Nozzle Diameter on Filament Strength and Gelation in Extrusion-Based 3D Printing with In Situ Ionic Crosslinking

**DOI:** 10.3390/biomimetics9100589

**Published:** 2024-09-29

**Authors:** Taieba Tuba Rahman, Al Mazedur Rahman, Zhijian Pei, Nathan Wood, Hongmin Qin

**Affiliations:** 1 Department of Industrial & Systems Engineering, Texas A&M University, College Station, TX 77843, USA; almazedurrahman@tamu.edu (A.M.R.); zjpei@tamu.edu (Z.P.); 2 Department of Biology, Texas A&M University, College Station, TX 77843, USA; woodn@tamu.edu (N.W.); hqin@bio.tamu.edu (H.Q.)

**Keywords:** bioink, 3D printing, coaxial nozzle, extrusion-based 3D printing, filament strength, gelation, in situ ionic crosslinking

## Abstract

This study systematically investigates the effects of the coaxial nozzle’s inner nozzle diameter on the strength and gelation of filaments produced via extrusion-based 3D printing with in situ ionic crosslinking. In this system, bioink (sodium alginate solution) was extruded through the outer nozzle, and the ionic crosslinking solution (calcium chloride solution) was extruded through the inner nozzle. The outer nozzle diameter was fixed at 2.16 mm, and the inner nozzle diameter was varied among 1.19, 0.84, and 0.584 mm. The results indicate that, as the inner nozzle diameter decreased, filament strength decreased, and filament gelation became poorer. These findings highlight the importance of optimizing inner nozzle diameter for improved filament strength and gelation in extrusion-based 3D printing with in situ ionic crosslinking.

## 1. Introduction

Bioprinting, a type of 3D printing, enables the fabrication of 3D biological constructs by precisely depositing bioink composed of cells and biomaterial [1]. Bioprinting can be used in tissue engineering to create functional tissue structures [2,3,4,5,6], in regenerative medicine to develop complex tissues and organs for transplantation [7,8,9,10], and in organ-on-a-chip models to replicate organ systems for drug testing and disease modeling [11,12,13]. Among various bioprinting techniques, extrusion-based 3D printing stands out due to its simplicity, cost-effectiveness, versatility in handling a broad spectrum of bioinks, and ability to fabricate large and intricate tissue constructs with high cell densities [14,15,16]. This technique builds 3D constructs layer by layer by extruding bioink through nozzles [16].

In extrusion-based 3D printing, the shape fidelity of printed constructs is crucial and can be achieved by crosslinking the bioink during or immediately after printing [17,18]. Shape fidelity is the ability of a printed construct to maintain its shape compared to the computer design [18,19,20]. Crosslinking refers to the process of forming chemical (covalent bonds) or physical bonds (physical interactions) between polymer chains within the bioink, transforming the bioink from a liquid state to a more solid-like gel state or hydrogel [21], thereby enhancing the mechanical stability and structural integrity of printed constructs [7,17]. A significant advancement in this domain is the use of in situ ionic crosslinking, particularly through a coaxial nozzle system [22]. This system, comprising an inner nozzle and an outer nozzle, usually extrudes the bioink through the outer nozzle and the ionic crosslinking solution through the inner nozzle. During the process of extrusion through the coaxial nozzle, when the bioink and the ionic crosslinking solution come into contact at the nozzle end, the bioink interacts with the ionic crosslinking solution to form a filament of hydrogel [18,23].

Researchers have recently designed and fabricated a 3D printing nozzle system with dual inlets and a single outlet to mix the material before extrusion for printing multi-material structures [24,25]. This system is different from the coaxial nozzle system (which had dual inlets and dual outlets) used in this study. In this study, bioink and crosslinking solutions came into contact at the nozzle end. Previously, in situ ionic crosslinking using a coaxial nozzle has been used for bioprinting with both animal cells and algae cells [18,23,26,27,28,29,30,31,32]. For example, Li et al. used in situ ionic crosslinking to print microchannel networks, with extruding hybrid bioink (composed of alginate, silk fibroin, and liver cancer cells) through the outer nozzle and extruding crosslinking solution (calcium ions and Pluronic F127) through the inner nozzle of the coaxial nozzle [26]. Rahman et al. printed 3D constructs with algae cells (Chlorella vulgaris) using in situ ionic crosslinking [18]. Table 1 summarizes some reported studies using extrusion-based 3D printing with in situ ionic crosslinking. Most reported studies have focused on the effects of bioink concentrations, crosslinking solution concentrations, and printing parameters (such as the printing speed, flow rate of bioink, and flow rate of crosslinking solution). Gao et al. showed that variations in inner nozzle diameter significantly impacted printed filament dimensions [23]. Sun et al. also showed that the inner nozzle diameter influenced the inner diameter of printed filaments [29].

Filament strength and gelation influence the structural integrity, precision, and functionality of printed constructs. When extruded filaments do not have sufficient strength, they cannot withstand gravitational forces, leading to sagging or deflection [33]. When extruded filaments are in an under-gelation condition, they remain in a more liquid-like state, leading to the fusion of upper and lower layers and preventing filaments from retaining a defined shape and structure [33,34]. For these reasons, ensuring adequate filament strength and proper gelation is essential in creating reproducible and accurate printed constructs. Despite the recognized importance of the inner nozzle diameter, there is a notable gap in understanding the specific impacts of the inner nozzle diameter on filament strength and gelation in extrusion-based 3D printing with in situ ionic crosslinking. The inner nozzle diameter directly affects the inter-nozzle gap of the coaxial nozzle system and the contact area between the bioink and crosslinking solution, both of which play a crucial role in the flow dynamics of the bioink and crosslinking solution, influencing the crosslinking efficiency, which is critical for the strength and gelation of the printed filaments. Therefore, it is important to understand the effects of the inner nozzle diameter on filament strength and gelation in extrusion-based 3D printing with in situ ionic crosslinking.

This study aims to fill a gap in the literature by systematically investigating the effects of the coaxial nozzle’s inner nozzle diameter on the strength and gelation of printed filaments via extrusion-based 3D printing with in situ ionic crosslinking. The findings of this study will contribute to the development of optimized extrusion-based 3D printing with in situ ionic crosslinking.

## 2. Materials and Methods

### 2.1. Preparation of Materials

#### 2.1.1. Preparation of Bioink (Sodium Alginate Solution)

Alginic acid sodium salt powder was obtained from Milipore Sigma (Product No. 180947, Sigma-Aldrich, St. Louis, MO, USA). This product is extracted from Phaeophyceae or brown algae. The ratio of mannuronic acid to guluronic acid (M/G ratio) is 1.56. The molecular weight of this alginic acid sodium salt powder is 120,000–190,000 g/moL. The viscosity of a 1% solution in water has a range of 15–25 millipascal-second (mPa.s). The sodium alginate solution was prepared following the procedure described in an earlier paper [18]. In brief, a 500 mL beaker with 100 mL of deionized water was put on a hot plate magnetic stirrer (Thermo Fisher Scientific, Waltham, MA, USA), and the stirrer was set to rotate at 800 rpm. Then, 3 g of alginic acid sodium salt powder (Sigma-Aldrich, Saint Louis, MO, USA) was slowly added to the beaker over 5 min to prevent clumping. The beaker was stirred by the hot plate magnetic stirrer at 60 °C for 2 h. Then, 200 µL of blue food dye (Wilton Great Value assorted food color and egg dye, Walmart, Bryan, TX, USA) was added to the beaker that contained the sodium alginate solution. The sodium alginate solution in the beaker was sterilized via autoclave at 121 °C for 20 min and was then stored at room temperature. The concentration of the final sodium alginate solution was 3% (*w*/*v*).

#### 2.1.2. Preparation of Crosslinking Solution (Calcium Chloride Solution)

The crosslinking solution was prepared following the procedure described in an earlier paper [18]. In brief, a 500 mL beaker was filled with 100 mL of deionized water and put on a hot plate magnetic stirrer (Thermo Fisher Scientific, Waltham, MA, USA), and the stirrer was set to rotate at 800 rpm. Then, 4 g of calcium chloride dihydrate powder (Sigma-Aldrich, Saint Louis, MO, USA) was slowly added to the beaker over 2 min. The beaker was stirred by the hot plate magnetic stirrer at room temperature for 30 min to ensure the total solvation of the powder in the water. The concentration of the final crosslinking solution was 4% (*w*/*v*). The concentration of 4% (*w*/*v*) was selected based on a previous study with alga cells using a similar experimental setup [18].

### 2.2. 3D Printing

**Figure 1:**
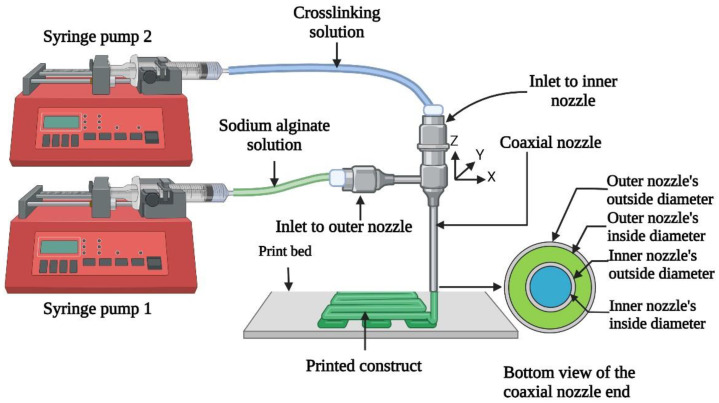
Illustration of extrusion-based 3D printing with in situ ionic crosslinking [18].

**Figure 2:**
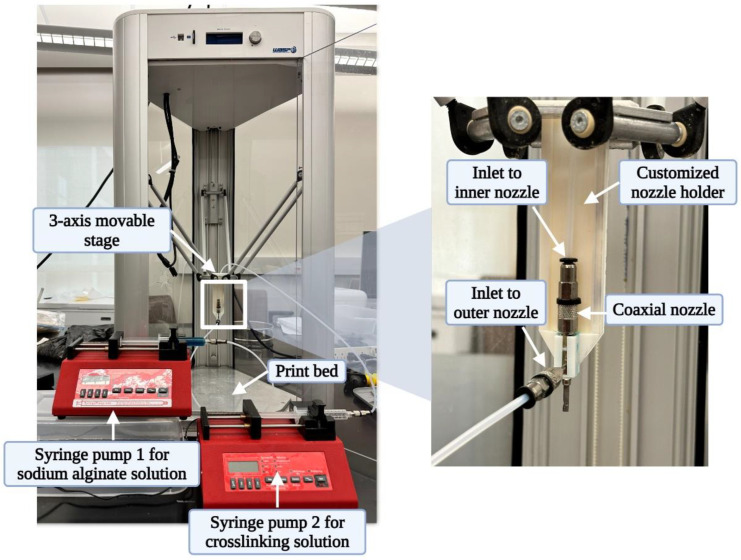
3D printing experiment setup with the Delta Wasp printer.

Extrusion-based 3D printing with in situ ionic crosslinking is illustrated in Figure 1 [18]. Figure 2 shows the 3D printing experiment setup. This setup included an extrusion-based 3D printer (Delta 2040, WASP, Massa Lombarda, Italy) with a 3-axis movable stage. A customized nozzle holder was attached to the movable stage to hold the coaxial nozzle (rame-hart instrument co., Succasunna, NJ, USA). The customized nozzle holder was designed using Autodesk Fusion 360 (version 2.0.16985) and fabricated from VeroPureWhite material (Stratasys Ltd., Way Eden Prairie, MN, USA) using the Stratasys J750 Polyjet 3D printer (Stratasys Ltd., Way Eden Prairie, MN, USA). The setup also had two syringe pumps (model NE-300, New Era Pump Systems Inc., Farmingdale, NY, USA). The bioink was extruded through the outer nozzle. The ionic crosslinking solution was simultaneously extruded through the inner nozzle.

The selection of specific nozzle diameters in this study was based on two requirements for the coaxial nozzle system. First, the outside diameter of the inner nozzle must be smaller than the inside diameter of the outer nozzle. Second, the inter-nozzle gap (the gap between the inside diameter of the outer nozzle and outside diameter of the inner nozzle) of the coaxial nozzle system must be adequate for fitting the inner nozzle within the outer nozzle and for maintaining the continuous extrusion of the bioink without clogging or causing excessive shear stress. In bioprinting, excessive shear stress can directly damage or rupture the cells embedded in the bioink [35]. Table 2 shows the values for the inner nozzle size, inside diameter of the inner nozzle, outside diameter of the inner nozzle, and inter-nozzle gap of the coaxial nozzle system. The inter-nozzle gap of the coaxial nozzle system is calculated by subtracting the outside diameter of the inner nozzle (*I_o_*) from the inside diameter of the outer nozzle (*O_i_*) and dividing the result by 2, as shown in Equation (1).

(1) $$Inte\mathrm{r-}nozzle\;gap=\frac{O_{i}-I_{o}}{2}$$

**Figure 3:**
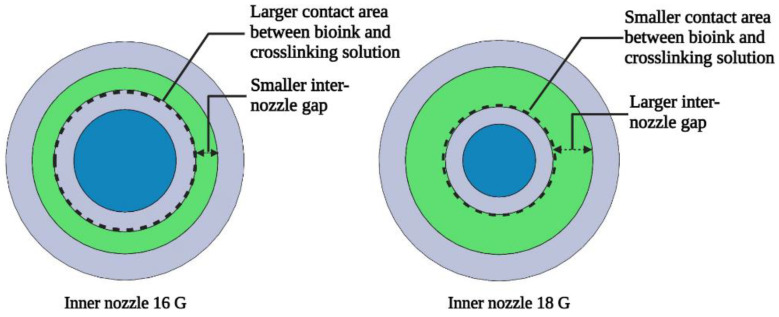
Differences in inter-nozzle gap and contact area (between bioink and crosslinking solution) for two different inner nozzle sizes in the coaxial nozzle system (with the outer nozzle size of 12 G).

In this study, the outer nozzle had an inside diameter of 2.16 mm (12 G), and the three inner nozzles used had inside diameters of 1.19 mm (16 G), 0.84 mm (18 G), and 0.584 mm (20 G), respectively. As shown in Table 2, for the inner nozzle with a size of 15 G, its outside diameter was 1.83 mm, and its wall thickness was 0.165 mm. Though this inner nozzle would fit within the outer nozzle, the inter-nozzle gap of the coaxial nozzle system was too small, leading to clogging or excessive shear stress. Similarly, the inner nozzle with a size of 14 G had an outside diameter of 2.11 mm, which was nearly equivalent to the inside diameter of the outer nozzle, leaving insufficient clearance to fit within the outer nozzle. Therefore, these two inner nozzles were not used in this study. Figure 3 illustrates the differences in inter-nozzle gap and contact area (between bioink and crosslinking solution) for two inner nozzle sizes (16 G and 18 G) in the coaxial nozzle system (with an outer nozzle size of 12 G). The selected nozzle diameters were suitable for the bioink and crosslinking solution used in this study. If different bioink and crosslinking solutions (that have different rheological properties) are used, then it is possible that different nozzle diameters might be needed.

In this study, the outer nozzle diameter (inside diameter of the outer nozzle) was fixed at 2.16 mm, and the inner nozzle diameter (inside diameter of the inner nozzle) was varied among 1.19, 0.84, and 0.584 mm. Thus, there were three experimental conditions. Under each experimental condition, three runs of printing experiments were performed to print three replicates used for the measurement of filament strength and three runs of printing experiments were performed to print three replicates used for the measurement of filament gelation. Table 3 shows the values of printing parameters that were kept constant. The flow rate of the syringe pump refers to the volume of fluid delivered per unit of time (µL/min). For syringe pumps, the flow rate is controlled by adjusting the speed at which the pump’s motor drives the syringe plunger forward, extruding the fluid. The printing speed is the speed at which the nozzle moves in the X-Y plane while printing [36]. The layer thickness refers to the thickness of each extruded layer.

### 2.3. Measurement of Filament Strength

**Figure 4:**
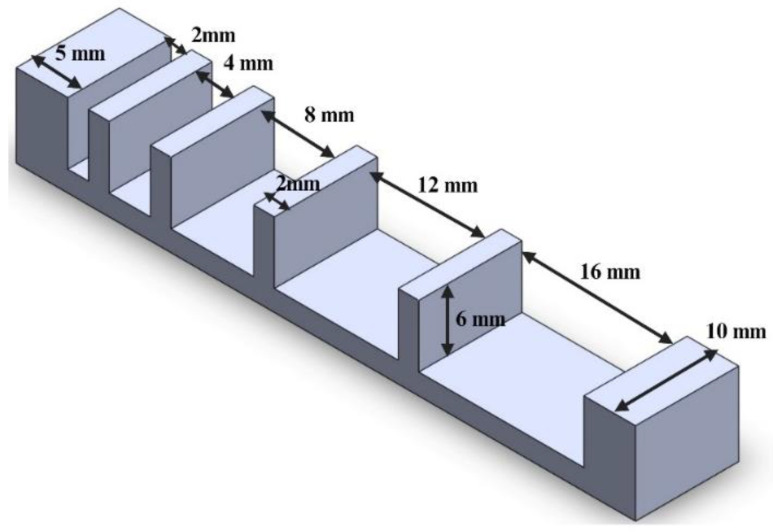
Platform used for measurement of filament strength.

Filament strength was quantitatively evaluated by the deflection of printed filaments over a platform (shown in Figure 4). Other researchers used this technique to study the effects of bioink composition on shape fidelity [34,37] and the effects of printing temperature on printability of alginate–gelatin bioink [33]. Enhanced filament strength allows for creating more robust and durable constructs that are critical in supporting tissue growth and regeneration. The constructs should maintain their structural integrity over an extended period of time, providing a stable environment for cell proliferation and differentiation [7]. In this study, the platform consisted of six pillars with an increasing distance (between two adjacent pillars) of 2, 4, 8, 12, and 16 mm. All six pillars had the same height of 6 mm and the same width of 10 mm. The two pillars on the ends had the same length of 5 mm, and the four pillars in between had the same length of 2 mm. The platform was designed using Autodesk Fusion 360 (version 2.0.16985) and fabricated from PLA (polylactic acid) material using the Ultimaker Cura S5 (Ultimaker, Utrecht, The Netherlands) 3D printer.

After printing, the platform with printed filament was kept at room temperature, under ambient light, for 5 min. Afterward, front-view images of the platform with the filament were taken using a Sony Alpha 9 II digital camera (Sony Corp., Minato, Tokyo, Japan). ImageJ software (version 1.53s) was used to analyze the images.

Deflection (*D_r_*) of the filament between each pair of adjacent pillars was determined using Equation (2).

(2) $$D_{r}=\frac{A_{t}-A_{a}}{A_{t}}$$

**Figure 5:**
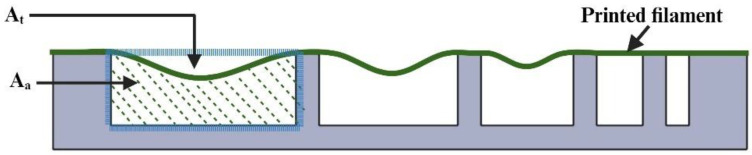
Printed filament over the platform, *A_a_* is the actual cross-section area and *A_t_* is the theoretical cross-section area.

*A_a_* is the actual cross-section area under the filament and between the two adjacent pillars. In Figure 5, A_a_ is the area indicated by the hatched green lines. *A_t_* is the theoretical cross-section area under an undeflected filament and between the two adjacent pillars. In Figure 5, *A_t_* is the area enclosed by the dashed blue lines. When the filament is unable to make a bridge between two adjacent pillars, the value of *A_a_* becomes zero, resulting in *D_r_* = 1. On the other hand, when the filament does not deflect and makes a straight bridge between two adjacent pillars, *A_a_* = *A_t_*, resulting in *D_r_* = 0. Deflection was calculated for each of the five spaces of the platform in each experimental run. Because there were three replicates of experimental runs under each experimental condition, the reported deflection value for each space of the platform in this paper is the average value of the three replicates.

### 2.4. Measurement of Filament Gelation

**Figure 6:**
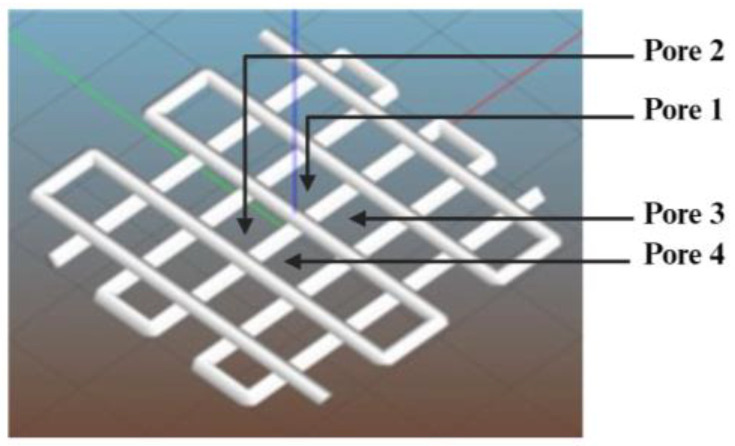
Construct design for the measurement of filament gelation.

Filament gelation was evaluated via the pore factor. Other researchers have used the pore factor to study the effects of bioink composition and printing temperature on printability [33,34,38]. In this study, a 2-layered construct (40 mm × 40 mm) in a grid pattern, as shown in Figure 6, was designed using Autodesk Fusion 360 (version 2.0.16985). Each grid square of the 2-layer construct is considered a pore.

After printing, the printed construct was kept at room temperature, under ambient light, for 5 min. Afterward, the top-view image of the printed construct was taken by using a Sony Alpha 9 II digital camera (Sony Corp., Minato, Tokyo, Japan).

Pore factor (*P_r_*) was calculated using Equation (3) [33,34,38].

(3) $$P_{r}=\frac{\pi }{4}\times \frac{1}{C}$$

where *C* is the circularity of a shape. It is a measure of how closely a shape resembles a perfect circle. The formula for circularity is as follows [34]:

C = 4πA/L^2^

where L stands for the perimeter of the shape and A stands for the area of the shape. A perfect circle has a circularity of 1, and a square has a circularity of *π*/4.

When the extruded filament is in a proper gelation condition, it would have a consistent filament width, resulting in regular grid squares or pores in the printed construct, and the value of the pore factor *P_r_* = 1. In an under-gelation condition, the extruded filament is in a more liquid-like state, causing the filament to spread and creating grid squares with a shape more like circular pores, leading to a *P_r_* value of less than 1. In contrast, when the extruded filament is in an over-gelation condition, it would have inconsistent width, resulting in irregular grids or pores in the printed construct, leading to a *P_r_* value greater than 1. ImageJ software (version 1.53s) was used to measure the area and perimeter of the pores from each printed construct. Four grid squares in the middle of the printed construct (labeled as 1, 2, 3, and 4 in Figure 6) were selected to calculate four pore factors from each of the printed constructs under each experimental condition. Eliminating the boundary squares of the printed construct can enhance the reliability of the data, as boundary squares often show inconsistent filament deposition. The reported pore factor for each printed construct in this paper is the average value of these four pore factors.

### 2.5. Statistical Analysis

A one-way analysis of variance (ANOVA) was performed on the experimental data to evaluate the statistical significance of the effects of the coaxial nozzle’s inner nozzle diameter on filament strength and gelation. Statistical software Minitab 18.0 was used to perform quantitative analysis, and OriginPro 8.5 was used to draw the graphs.

## 3. Results and Discussions

### 3.1. Effects on Filament Strength

**Figure 7:**
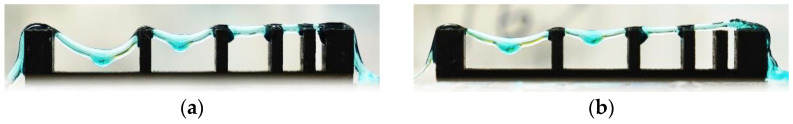
Printed filaments over the platform for the measurement of filament strength. (**a**) Inner nozzle diameter of 0.84 mm; (**b**) inner nozzle diameter of 1.19 mm.

Figure 7 shows front-view images of printed filaments over the platform for two inner nozzle diameter values (1.19 and 0.84 mm, respectively) of the coaxial nozzle. The printing was not successful for the inner nozzle diameter of 0.58 mm. During printing with this diameter of 0.58 mm, the extruded filament failed to adhere to the platform, causing the nozzle to drag the filament along the printing path. This is likely due to the smaller inner nozzle diameter, which leads to poor crosslinking. When the syringe pump maintains a constant flow rate, it moves the same volume of fluid through the nozzle per unit of time. However, as the inside diameter of the inner nozzle decreases, the resistance to flow increases significantly, according to the Hagen–Poiseuille equation, as shown in Equation (4) [39].

(4) $$Q=\frac{\pi \Delta P{\left(\frac{d}{2}\right)}^{4}}{8\eta L}$$

In this equation, Q represents the flow rate, ΔP is the pressure difference across the nozzle, d is the inside diameter of the nozzle, η is the dynamic viscosity of the fluid, and L is the length of the nozzle. This equation indicates that the pressure (ΔP) required to maintain a given flow rate (Q) increases sharply as the nozzle diameter (d) decreases. The increase in pressure affects the flow dynamics and can lead to uneven or disrupted extrusion in the crosslinking solution. As the pressure rises, the fluid flow may become uneven or turbulent, especially near the nozzle exit, causing fluctuations in flow rate and making the extrusion of the crosslinking solution less consistent. Additionally, the elevated pressure can create backpressure within the system, resisting the smooth flow of the crosslinking solution and resulting in a smaller effective amount being extruded. Consequently, this disturbance in flow leads to reduced crosslinking efficiency when a smaller inside diameter of the inner nozzle is used.

**Figure 8:**
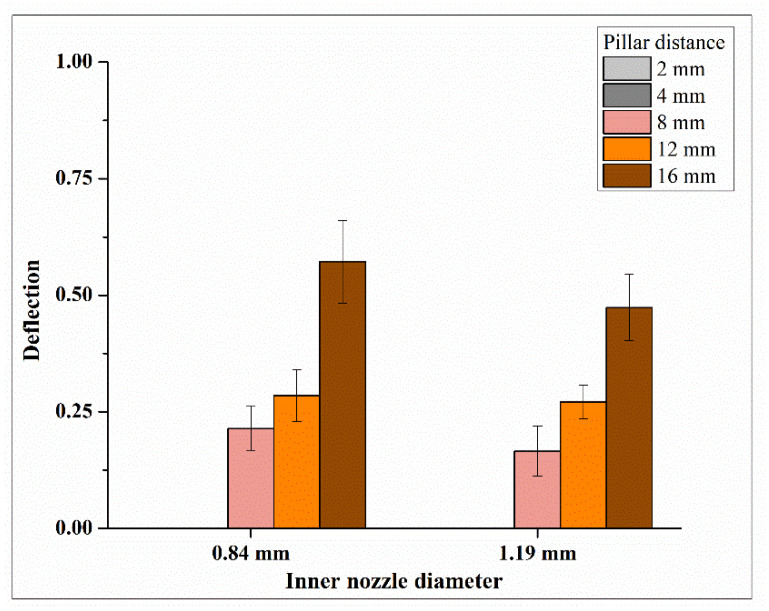
Relationship between inner nozzle diameter and deflection of printed filaments.

Figure 8 shows the relationship between the inner nozzle diameter and deflection of printed filaments. Each data point is presented as mean ± standard deviation. Both Figure 7 and Figure 8 show that, as the inner nozzle diameter increased from 0.84 to 1.19 mm, the deflection of the printed filament decreased, and, therefore, the filament strength increased. A possible explanation is as follows. A larger inner nozzle diameter results in a larger inner diameter in the hollow filament, while the outer diameter of the filament remains relatively unchanged [23,29]. This leads to a smaller wall thickness in the printed filament, which allows for more effective radial diffusion and a higher concentration of crosslinking ions within the alginate matrix, resulting in better crosslinking through the thickness of the printed filament and improved filament strength. In this study, a 3% sodium alginate solution was used as the bioink, while a 4% calcium chloride (CaCl_2_) solution was used as the crosslinking solution. These concentrations were selected from the reported studies [18,31]. During the coaxial extrusion process, the crosslinking solution was extruded through the core section of the filament, allowing Ca^2^⁺ ions to diffuse across the wall thickness of the hollow filament. The gelation of sodium alginate solution occurred via a crosslinking mechanism, where the divalent Ca^2^⁺ ions replaced the Na⁺ ions at the G-blocks of sodium alginate, leading to gel formation. A reported study [29] showed that the diffusion-dependent gelation of alginate is influenced by concentrations of both alginate and CaCl_2_. For instance, in the case of a 4% alginate solution, different crosslinker concentration (2–5% CaCl_2_) would result in different dimensions of printed filaments due to different diffusion rates of Ca^2^⁺ ions throughout the filament wall thickness. At a lower concentration of CaCl_2_ (e.g., 2%), only a small portion of the extruded alginate was crosslinked, which could be compensated by increasing the CaCl_2_ concentration. The same study also investigated the effects of different alginate concentrations (3–6%) while maintaining a fixed CaCl_2_ concentration of 4%. It was observed that higher alginate concentrations reduced the diffusion rate of Ca^2^⁺ ions and altered the filament wall thickness. Specifically, at 4% CaCl_2_, the wall thickness of the hollow filament was larger with a 3% sodium alginate solution compared to a 4% sodium alginate solution.

Figure 8 shows that the pillar distance also affected deflection of printed filaments (as the distance between adjacent pillars increased, deflection increased). For pillar distances of 2 and 4 mm, the printed filament did not deflect and made a straight bridge between two adjacent pillars, resulting in a deflection value of 0 for both inner nozzle diameters. Therefore, only the deflection values for pillar distances of 8, 12, and 16 mm for the platform in each experimental run were used for one-way ANOVA.

Table 4 presents the ANOVA results of the effects on filament strength at the pillar distance of 16 mm. The ANOVA results indicate a *p*-value of 0.208 at the pillar distance of 16 mm, which is lower than the *p*-values at pillar distances of 8 mm (*p*-value of 0.309) and 12 mm (*p*-value of 0.740). Therefore, pillar distances of less than 16 mm were not large enough to reveal the deflection differences caused by the change in inner nozzle diameter.

### 3.2. Effects on Gelation

**Figure 9:**
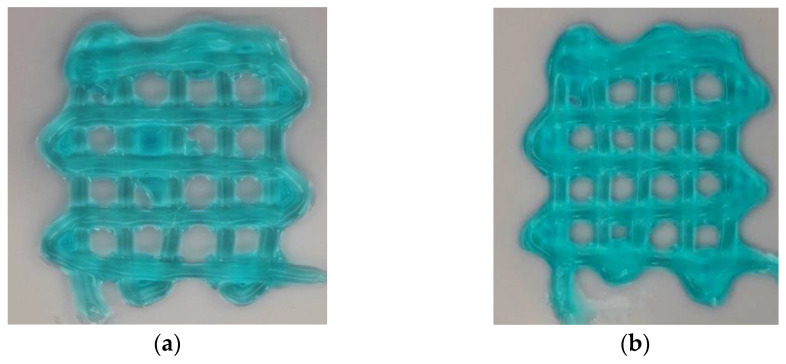
Printed constructs for the measurement of filament gelation. (**a**) Inner nozzle diameter of 0.84 mm; (**b**) inner nozzle diameter of 1.19 mm.

**Figure 10:**
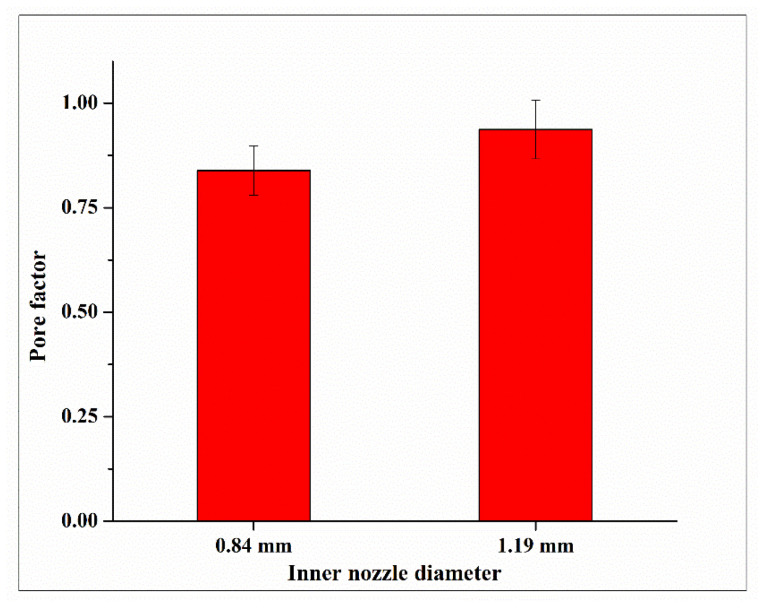
Relationship between inner nozzle diameter and pore factor in printed constructs for the measurement of filament gelation.

Figure 9 shows top-view images of printed constructs for two inner nozzle diameters (0.84 and 1.19 mm) of the coaxial nozzle. For the inner nozzle diameter of 0.584, during printing, the coaxial nozzle dragged the printed filament along the printing path, failing to fabricate a 2-layered construct due to the under-gelation of the printed filament. Under-gelation occurs when the crosslinking of the bioink is insufficient, resulting in a filament that is too fluid to maintain its shape. Figure 10 shows the relationship between the inner nozzle diameter and pore factor of printed constructs, and each data point is presented as mean ± standard deviation.

Both Figure 9 and Figure 10 show that, when the inner nozzle diameter of the coaxial nozzle increased from 0.84 to 1.19 mm, the pore factor increased. This implies that a larger inner nozzle diameter improves filament gelation. This is likely because the nozzle with a larger inner nozzle diameter results in a smaller inter-nozzle gap in the coaxial nozzle system and a larger contact area between the bioink and crosslinking solution, which allows for more effective radial diffusion and a higher number of divalent cations of calcium ions within the alginate matrix. This leads to better crosslinking and improved filament gelation.

Table 5 presents the ANOVA results for the effects on filament gelation. The average of the four pore factors of the printed construct (in the middle of the printed construct and labeled as 1, 2, 3, and 4 in Figure 5) in each experimental run was used for one-way ANOVA. The ANOVA results indicate a *p*-value of 0.137.

## 4. Conclusions

This paper presents an experimental study investigating the effects of the coaxial nozzle’s inner nozzle diameter on filament strength and gelation in extrusion-based 3D printing with in situ ionic crosslinking. The inner nozzle was used for extruding crosslinking solution (calcium chloride solution), while the outer nozzle was used for extruding bioink (sodium alginate solution). For the coaxial nozzle setup, the outside diameter of the inner nozzle must be smaller than the inside diameter of the outer nozzle. The coaxial nozzle diameter ratio refers to the ratio between the inside diameter of the inner nozzle and the inside diameter of the outer nozzle. When the inner nozzle diameter is larger, the coaxial nozzle diameter ratio is higher. The results show that as the coaxial nozzle diameter ratio increased, both filament strength and gelation increased.

In this study, filament strength was evaluated by the deflection of printed single-layer filaments over a platform, while filament gelation was evaluated by the pore factor obtained from the top view of the printed two-layered construct with a grid pattern. However, the findings are useful for multi-layered bioprinting. Understanding how the inside diameter of the inner nozzle influences filament strength and gelation allows researchers to adjust the diameters of the coaxial nozzle system to obtain desired properties in more complex 3D printed constructs. Additionally, the authors’ previous work utilized the same extrusion-based 3D printing with in situ ionic crosslinking to create a multi-layered construct with alga cell-laden bioink [18].

As the first of a series of studies, the objective of this study was to investigate the effects of the inside diameter of the inner nozzle. The authors plan to study other processing parameters (such as the scanning speed, bioink composition, crosslinking solution concentration, and rates of bioink and crosslinking solution) in their future research and publish the results in separate papers. Future research topics also include conducting additional quantitative experiments such as stretching and bending to determine the conditions of failure. Another future research topic regarding coaxial nozzle design is to embed animal or alga cells in the bioink and determine the effects of the coaxial nozzle’s inner nozzle diameter on cell viability. Cell viability is defined as the ability of cells to survive and maintain metabolic activity under given conditions [40,41].

## Figures and Tables

**Table 1:** Reported studies using extrusion-based 3D printing with in situ ionic crosslinking.

Parameter	Response Variable	Reference
Bioink concentration and flow rate, crosslinking solution concentration and flow rate, inner nozzle diameter, and printing speed	Diameter and shape of printed filaments, filament fusion, ultimate strength and failure strain of printed constructs	[23]
Bioink concentration and flow rate, and crosslinking solution concentration and flow rate	Dimensions of printed filaments	[28]
Crosslinking solution flow rate and inner nozzle diameter	Inner diameter of printed filaments	[29]
Bioink concentration and flow rate, and crosslinking solution concentration and flow rate	Dimensions of printed filaments	[31]
Bioink concentration	Shrinkage, swelling, and dimension of printed filaments	[32]

**Table 2:** Values for the inner nozzle size, inside diameter of the inner nozzle, outside diameter of the inner nozzle, and inter-nozzle gap of the coaxial nozzle system (with an outer nozzle size of 12 G).

Inner Nozzle Size (Gauge)	Inside Diameter of the Inner Nozzle (mm)	Outside Diameter of the Inner Nozzle (mm)	Inter-Nozzle Gap of the Coaxial Nozzle System (mm)
14 G	1.60	2.11	0.025
15 G	1.37	1.83	0.165
16 G	1.19	1.65	0.255
18 G	0.84	1.24	0.46
20 G	0.584	0.889	0.6355

**Table 3:** Printing parameters that were kept constant and their values.

Parameter	Unit	Value
Flow rate of the syringe pump for sodium alginate solution	µL/min	600
Flow rate of the syringe pump for crosslinking solution	µL/min	600
Coaxial nozzle’s outer nozzle diameter	mm	2.16
Printing speed	mm/s	8
Layer thickness	mm	1.6

**Table 4:** ANOVA for effects on filament strength (pillar distance of 16 mm).

Source of Variance	Degree of Freedom	Adj Sum of Squares	F-Value	*p*-Value
Inner nozzle diameter	2	0.014	2.256	0.208
Error	6	0.026		
Total	8	0.040		

**Table 5:** ANOVA for effects on filament gelation.

Source of Variance	Degree of Freedom	Adj Sum of Squares	F-Value	*p*-Value
Inner nozzle diameter	2	0.014	3.453	0.137
Error	6	0.017		
Total	8	0.031		

## Data Availability

The authors confirm that the data to support the findings of this study are available within the article or upon request from the corresponding author.
